# History of Trace Analysis

**DOI:** 10.6028/jres.093.013

**Published:** 1988-06-01

**Authors:** H. A. Laitinen

**Affiliations:** Department of Chemistry, University of Florida, Gainesville, FL 32611

In the era of classical analysis when major and minor constituents of materials such as rocks and ores were determined by gravimetric and titrimetric methods, a measure of the quality of an analysis was the closeness to which the summation of constituents approached 100%. Trace constituents were considered to be those known to be present but in amounts so small that they made no appreciable contribution to the summation. An early authority was Hillebrand [1], who in 1919 wrote his classic book “Analysis of Silicate and Carbonate Rocks” and used the word “trace” to designate constituents present below the limit of quantitative determination, which meant below 0.01 or 0.02 percent. Sandell [2], in his 1944 book “Colorimetric Determination of Traces of Metals,” considered major constituents to be those present in amounts greater than 1%, minor constituents to be those present in amounts between 0.01 and 1%, and trace constituents those below 0.01%.

The modern definition of “trace” is more flexible, as illustrated by a quotation from a 1965 book, “Trace Analysis” edited by George Morrision [3]: “The connotation of the term “trace” varies with the background or interests of the reader.” In that book, the upper limit was considered to be about 100 ppm by weight, and the term “ultratrace” was used for constituents below 1 ppm. To quote further, “any sharp division is, of course, superfluous, and will depend on the nature of the sample to be analyzed, the analytical technique employed, and the analyst.”

For trace analysis to emerge as a specialty in its own right, two conditions had to be met: specific needs and applicable methods. Qualitative methods in general emerged much earlier than quantitative ones. Quite a few qualitative tests and even a few quantitative methods of great sensitivity existed before the turn of the century, but they remained largely unused as interesting curiosities until a need arose. The decade of the 1940s represented a watershed in creating a variety of new demands for analytical methods of exceptional sensitivity and difficulty. World War II had quite a stimulating effect with respect to new needs, but it also stifled free publication for several years, with the result that shortly after the end of the war in 1945, there was a release of enormous amounts of previously classified material for publication. Methods and instrumentation developed to solve specific problems now became available for wider application.

It is now convenient to consider five periods in history—(1) antiquity to the beginning of modern chemistry late in the 18th century, (2) late 18th century through the 19th century, (3) the period from 1900 to 1939, (4) the decade of the 1940s, and (5) the period from 1950 to the present.

## Period 1, Antiquity to Late 18th Century

Probably the earliest example of trace analysis is fire assay or cupellation, to which several references are made in the Old Testament.

Szabadvary [4] states that “Pliny records the use of extract of gall nuts as a chemical reagent when soaked on papyrus. Adulteration of copper sulfate with iron sulfate could be detected by the papyrus becoming black when dipped in the sulfate solution.” This test for iron, first described in 61 A.D., emerges again in 1576 (Gesner) and 1597 (Libavius) [5]. Apparently the first use of gall-nut powder for a quantitative analysis was by Robert Boyle to estimate the amount of iron in natural waters (1684). The limit of detection was estimated to be 1 part in 600 or 160 ppm. This reagent was also used for copper. Boyle suggested other plant extracts as reagents but these did not prove to be reliable. He introduced a new reagent he called “volatile sulphureous spirit,” later identified by Szabzadvary as hydrogen sulfide, which did not receive attention as an analytical reagent for another century. Boyle is credited with the first use of the term “chemical analysis” as we know it today in 1654, and with the introduction of litmus as an acid-base indicator.

The phlogiston era, from the late 17th to the late 18th century, was essentially barren from the viewpoint of trace analysis. An exception is the work of Marggraf (1709–82), who used the Prussian Blue test for iron, a flame test to distinguish between sodium and potassium salts, and the microscope as an analytical instrument. Another important chemist of this era was Torbern Bergman (1733–84), who wrote the first analytical textbook (1780) and originated analytical chemistry as a distinct branch of chemistry [5].

## Period 2, Late 18th Century to 1899

Modern chemistry began to flourish with the abandonment of the phlogiston theory, but for a long time trace analysis remained of interest for only a few constituents of special value or special effects, such as imparting color, taste, or odor to drinking water.

Colorimetry developed relatively early in the primitive form of visual comparison of the intensity of color of an unknown in a cylindrical tube with that of a series of standards of known concentration. Some early examples are the estimation of iron or nickel in a cobalt ore (Lampadius, 1838), copper via the ammonia complex (Jacquelain, 1846), iron via the thiocyanate complex (Herapath, 1852), titanium via hydrogen peroxide (1870), hydrogen sulfide via methylene blue (1883), and silica via molybdosilicic acid (1898) [5]. The Duboscq colorimeter (1854) represented a breakthrough in permitting the relative light paths of the standard and unknown to be varied mechanically until an equal intensity is observed. This instrument remained in common use until the introduction of the photoelectric colorimeter in the late 1920s. Some of these early methods had limits of detection and determination in the microgram range and could be called trace analytical methods, but the need for such methods remained rather specialized and limited throughout the 19th century and the early 20th century.

An outstanding development of the 19th century was atomic emission spectroscopy, introduced by Bunsen and Kirchhoff in 1860 [6]. Talbot, in Scotland, had used a spectroscope to observe flame colors as early as 1826, and had suggested the use of this method for trace analysis, but it attracted little interest for several decades. It is interesting to speculate on the reason for this long gap. Talbot had observed the difference between lithium and calcium salts and had noted the great sensitivity of the method, but he did not pursue the scope of the method or contribute to the fundamental understanding of the subject. Bunsen and Kirchhoff, on the other hand, showed the reason for the Fraunhofer lines of the solar spectrum which had been observed as early as 1814, made quantitative studies of emission and absorption, and clearly associated specific emission lines with elements rather than compounds. The Bunsen burner also was a simple and practical emission source. Their method rapidly saw many applications, including the discovery of several elements [Cs, Rb (1860), T1 (1861) and In (1864)].

However, apart from Bunsen’s laboratory, the method did not see general use for several decades, even for qualitative purposes. Slavin [7] has traced several reasons for this neglect: (a) Flame sources showed poor sensitivity for metals other than the alkali metals and alkaline earths; (b) While electrical discharges had long been known to produce spectra of almost all metals, there were not convenient means of providing electric current in Bunsen’s day; (c) There were no wavelength tables available to interpret the complex arc and spark spectra; and (d) Photographic recording was not in general use until later. To these reasons Winefordner added the general inertia of the scientific community. As recently as 1910, H. Kayser stated “There is little prospect that in the future qualitative analysis will apply spectroscopic methods to a large extent … I have come to the conclusion that quantitative spectroscopic analysis has shown itself to be impractical.” Slavin remarks: “Thus by 1920 all the conditions needed for a system of chemical analysis by spectroscopy existed. We had excellent instruments, good photographic emulsions, a power distribution network, and basic theory. However, chemists were very slow to take advantage of this powerful tool, even for simple qualitative identifications. They still relied on the classical instruments, the test tube, the blowpipe, and the nose.” It was left for physicists, astronomers, and others to develop the method until the 1930s.

Electroanalytical chemistry can be traced back to 1833, when Faraday discovered the laws of quantitative electrolysis. Electrogravimetry, which could in some cases be applied to trace analysis, dates back to 1864. Real advances, however, were not to come about until the emergence of solution physical chemistry around the turn of the century.

O’Haver [8] has traced the development of luminescence spectrometry (the measurement of fluorescence and phosphorescence) in analytical chemistry. Fluorescence has been recognized since 1833, when Brewster described the emission of red light by an alcoholic extract of green leaves (chlorophyll) and described the phenomenon as “dispersion” [9]. The term “fluorescence” was introduced by Stokes, who in 1852 first recognized that the emitted light was of a longer wavelength than the exciting radiation, and who proposed the use of fluorescence as an analytical tool in 1864. The first use of fluorescence in trace analysis was the determination of aluminum by means of the fluorescence of the morin complex by Goppelsroder in 1867 [10]. Until 1920, fluorescence intensities were estimated by visual comparator methods, and further development awaited the introduction of more advanced instrumentation.

A closely related method is phosphorescence, which is characterized by a time delay in the emission. This phenomenon has been recognized since 1568, when Cellini described a luminescent diamond [11]. A great many phosphors were discovered during the 17th and 18th centuries, but little progress was made until Becquerel devised the first phosphoroscope in 1858 and established the exponential decay law in 1861. Quantitative trace applications, however did not emerge until the 1950s.

## Period 3, 1900–1939

The whole nature of analytical chemistry underwent a profound change when the principles of physical chemistry began to be applied systematically to the understanding of analytical procedures. A highlight was in 1894, when Wilhelm Ostwald, one of the leading physical chemists of the day, published a book entitled “Die Wissenschaftlichen Grundlagen der Analytische Chemie.” Ostwald showed how ionic equilibria could be applied to acid-base and precipitation reactions, how precipitates undergo recrystallization upon standing (Ostwald ripening), etc.

Oddly enough, he did not mention the Nernst Equation, which had been published in 1889 [12] while Nernst was in Ostwald’s laboratory. Nevertheless, Kolthoff [13] has characterized Ostwald together with Gibbs, van’t Hoff and Arrhenius, as “the founders of physical chemistry, and, indirectly, of scientific analytical chemistry.” It was Salomon [14], in Nernst’s laboratory, who in 1897 performed the first “galvanometric” titration, the forerunner of the modern biamperometric titration. Nernst and Merriam [15], in 1905, established the basis of steady state voltammetry using stationary and rotating electrodes and interpreted them on the basis of the Nernst diffusion layer. These methods were not clearly understood until later, when polarography had been developed, and they did not receive application until the 1940s.

Sorensen’s development of the concept of the pH in 1909 led to a direct application of the Nernst equation to trace analysis [16]. The development of the glass electrode as a pH electrode by Haber and Klemensiewicz [17], also in 1909, was later to revolutionize pH measurements as soon as reliable electronic instruments became available for measurements using the high impedance membranes. There might be some question about the inclusion of pH as trace analysis, but considering the fact that the ion selective electrodes are based on the same principle this inclusion appears appropriate.

Potentiometric titrations also originated with Nernst. A great many analytical applications were made by pioneers such as Erich Müller and I. M. Kolthoff during the 1920s, but the emphasis was on accurate and selective titrations rather than on trace analysis.

The discovery of polarography in 1922 [18] by Heyrovsky was landmark because it introduced a new approach to trace analysis. During the period 1922–39, a great many publications on classical polarography appeared, mainly in the *Collections of the Czechoslovak Chemical Communications*, Many trace analytical applications were described for inorganic, organic, and biological systems. Although Kolthoff and his students had been involved in polarographic research since 1935, no publications emerged from his school until 1939 [19]. European laboratories were making many applications, but the only commercial apparatus available was the original Nejedly instrument introduced in 1925 in Prague and no English language book was available. The method required considerable investment in effort for sufficient understanding, and did not lend itself to empirical applications without this understanding. Applications were relatively few in the U.S.A. through the 1930s, but the picture was soon to change with the appearance of the Kolthoff-Lingane book “Polarography” in 1941 and with the introduction of U.S.-made instrumentation.

The most sensitive trace analytical method cited by Sandell, op. cit. is the isolation of a bead of gold from two liters of sea water followed by its microscopical measurement. This method, described by Fritz Haber in 1927 [20], was used to estimate that sea water contained variable amounts of gold, on the order of 10^−10^%, depending on the locality. The accepted average value is 4×10^−10^% or 4 *μ*g/L.

Some of the early colorimetric methods have already been mentioned. With the development of the photoelectric colorimeter and the spectrophotometer and the increasing demand for trace methods during the 1930s and 40s, and with the increasing knowledge about solution equilibria involving coordination compounds, a great many sensitive trace methods emerged. Especially noteworthy is the dithizone method, based on selective extraction of trace metals as dithizone complexes to enhance both the selectivity and sensitivity of the methods. Sandell [2] gave a practical limit of about 0.1 ppm for quantitative colorimetric determinations in solid samples.

Fluorescence methods were stimulated by the introduction of a photoelectric fluorometer by Jette and West in 1928 [21]. Cohen, in 1935, described a simple fluorometer and depicted a typical analytical calibration curve [18]. Finally, invention of the photomultiplier in 1939 greatly improved the sensitivities of fluorescence methods. The first complete commercial fluorescence spectrometer was introduced by Aminco in 1955.

A special form of luminescence is observed when certain metal oxides containing trace quantities of activating elements are placed at the outer edge of a hydrogen diffusion flame. This phenomenon was observed as early as 1842 by Balmain and termed *candoluminescence* by Nichols in 1928. Its use in qualitative analysis dates back to Donau in 1913, but its use as a quantitative trace method is primarily due to Townshend and Belcher, beginning in 1972 [23]. It has not seen extensive application, evidently because of the inconvenient sample preparations required.

Although catalyzed reactions have been long recognized and used as the basis of sensitive qualitative tests, the first quantitative use of reaction rates in trace analysis appears to be the work of Sandell and Kolthoff [24] in 1934, who showed that the rate of the Ce(IV)-As(III) reaction was proportional to the concentration of iodide present as a catalyst, and used the rate measurement to estimate iodide concentrations down to 20 ppb.

## Period 4, the 1940s

The decade of the 1940s represents a special time in the history of trace analysis because the outbreak of World War II in September 1939 suddenly cut off a great deal of international communication. Even domestic communication was impeded because of the secrecy of several wartime research programs. These research programs introduced an urgent need for trace analytical methods of a wide variety. In the years immediately following the end of the war in 1945, a great surge of publication occurred. Fortunately, the ACS had foreseen the revolutionary changes occurring in analytical chemistry, and had prepared for the flood of publications. In 1943, Walter J. Murphy became editor of *Industrial and Engineering Chemistry* and of its *Analytical Edition*. He soon brought in L. T. Hallett as an associate editor, and they began to lay plans for a separate analytical journal. Ralph H. Müller began a column on Instrumentation in 1946, a new format had been adopted by 1947, and in 1948 the new name of *Analytical Chemistry* became fully operational.

Upon the outbreak of World War II, the delivery of the Nejedly Polarograph was cut off, and the E. H. Sargent company was granted the right to market U.S.-made instruments under the same name. The first U.S.-made photographic-recording instruments of 1940 were later replaced by pen-and-ink instruments introduced by Sargent and by Leeds and Northrup. For some 25 years, polarography had dominated electroanalytical chemistry, but beginning in the 1940s other microelectrode techniques began to supplant and replace classical polarography.

The long time interval between the first discovery of the principles of steady state voltammetry and of amperometric titrations and their modern usage has already been noted. There are other interesting gaps of this sort. Coulometry could be said to date back to Faraday, but it did not emerge as a modern analytical technique until 1938, when Szebelledy and Somogyi [25] introduced coulometric titrations at constant current. The companion technique of coulometric analysis at controlled potential was pioneered by Hickling’s [26] development of the electronic potentiostat in 1942, and Lingane’s [27] applications of exhaustive electrolysis in the late 1940s. These applications were hampered by the lack of an electronic coulometer. The modern form of this instrument, based on storing a portion of the electric charge in a capacitor and measuring the voltage drop across the capacitor as a function of time, could not be developed until advances in dielectric materials and electronic measurements had occurred.

Of the wartime programs, the best known is the nuclear energy program, which put severe demands upon trace analysis capabilities. Not only were materials such as graphite needed in unheard-of purity levels, but methods were needed for elements that did not even exist in nature, and for elements in matrices of exceptional complexity, such as fission products. Methods were needed for accurate isotope ratios, and for extremely small amounts of elements of unknown chemistry, the transuranium elements.

Less generally recognized are the other classified programs of the era. The antimalarial program required analytical methods for new drugs in blood plasma at concentration levels that kept decreasing as the drugs improved. Here extraction and fluorescence methods sensitive to ppb levels were devised. Another wartime effort was the synthetic rubber program which involved emulsion polymerization for the first time in the U.S., and which brought demands for trace analysis methods not only in the emulsion system but for the raw materials and products. In the area of chemical warfare, trace methods were needed for known chemical agents as well as for new ones being developed.

These wartime demands spawned a reexamination of many existing trace methods, the adaptation of old methods to new problems, and the creation of entirely new approaches, Instrumentation often had to be improved to meet new demands, but electronics was still in the era of vacuum tubes and the digital computer had not yet been developed, so much remained to be done in the postwar period. Let us examine a few of the wartime advances.

In 1939, mass spectrometry was still in a relatively primitive state as far as trace analysis is concerned [7]. The early instruments of J. J. Thomson, A. J. Dempster, and F. W. Aston were primarily used to identify nuclides of the various elements. A. O. C. Nier, in the 1930s, greatly refined the quantitative measurement of isotope ratios, and early in the nuclear program designed instruments for isotopic analysis of uranium and hydrogen. Organic MS analysis was developed primarily for hydrocarbon analysis and applied during the wartime polymer program. In 1945, the fragmentation patterns of aliphatic hydrocarbons were published by H. W. Washburn et al. of the Consolidated Engineering Co., which introduced the first commercial MS instrument shortly thereafter.

Ion exchange had been recognized since 1850, but few analytical applications had been made by 1939. The first synthetic ion exchangers appeared in the mid-30s, but the real impetus for analytical separations came during the nuclear program when rapid rare earth separations became necessary. These developments, both at Oak Ridge under G. E. Boyd and at Iowa State under F. H. Spedding, were published in 1947 [28,29] and led to a large number of analytical applications. The polystyrene-based ion exchange resins, introduced in 1944, form the basis of modern applications.

Infrared spectrometry, in 1939, was still largely a specialized structural tool although a few analyses of major and minor constituents had been reported. A key publication by Norman Wright of Dow in 1941 showed the possibility of organic analysis by IR [7]. The wartime polymer program gave a great impetus for applications such as monitoring hydrocarbon purity and measuring side vinyl groups in elastomers. Commercial instrumentation, beginning in 1942 with the Beckman IR-1, and in 1944 with the Perkin-Elmer Model 12A, moved infrared gradually from the physics laboratory into analytical applications. Real stimulus came later, with the introduction of the Perkin-Elmer Model 137, a relatively low cost bench top instrument, which has been succeeded by a series of instruments designed for general use.

In a similar way, UV spectroscopy was stimulated to emerge as an everyday analytical method through the introduction of the Beckman DU instrument in 1941 [7]. Publication of many wartime applications was delayed until the late 1940s.

Atomic emission spectroscopy, as mentioned above, remained largely a qualitative method until the introduction of methods for comparison of line intensities on photographic plates were worked out in the 1920s and 30s. These methods included the log sector and step sector rotating disks and the microphotometer. The atomic emission method remained relatively cumbersome and inexact until the direct reading spectrometer became a reality in the late 1940s. For this to happen, the photomultiplier tube and associated electronics had to be developed. Many later developments have involved multichannel capabilities and computerized data processing.

Electroanalytical methods likewise found many trace applications stimulated by wartime needs. For example, amperometric titration methods found application for monitoring mercaptan levels in emulsion polymerization systems, and in coulometric titrations of trace arsenic at micromolar concentration levels. Linear sweep and cyclic voltammetry were studied independently in several countries although publication did not emerge until after the war. Several electroanalytical methods were hampered by instrumental limitations which were gradually overcome in postwar years by improvements in oscilloscopes, development of solid state electronics, and the microcomputer.

## Period 5, 1950 to Present

The invention of the transistor in 1947 proved to be critical in revolutionizing instrumental approaches to trace analysis. Not only were solid state electronic devices more reliable, stable, sensitive, and less expensive than their vacuum tube counterparts but they consumed far less power and were capable of miniaturization. The microcomputer became so much cheaper and smaller that it became practical to incorporate data processing elements into individual instruments. The laser and fiber optic techniques have permitted miniaturization of a variety of optical methods. The recent history of many trace methods has involved the use of long known principles of physics and rendering them practical for analytical applications through modern instrumentation.

Electroanalytical chemistry since 1950 has moved in several directions, including (a) ion selective electrodes, (b) other electrochemical sensors, and (c) combinations of electrochemical and optical techniques. These will be considered in turn.

a. Ion selective electrodes in their modern forms are relatively recent developments, beginning in the 1960s. As early as 1923, Horovitz [30] showed that glass electrodes responded to ions other than the hydrogen ion, e.g., sodium, potassium, silver, and zinc ions especially at lower acidities. Eisenman et al. [31] pioneered in the theoretical interpretation of mixed response of glass membranes to cations.

A few solid state membrane electrodes were described by Trümpler [32] in 1921, Tendeloo [33] in 1936, and to Kolthoff and Sanders [34] in 1937. No important applications were made until the work of Pungor et al. in the 1960s [35]. Pungor described composite electrodes of solid particles imbedded in silicone rubber which acted as solid membranes. A landmark discovery was the solid state fluoride sensing electrode of Frant and Ross [36] in 1966. Another landmark was the introduction of liquid membrane sensors. As early as 1933 Beutner [37] studied water-immiscible organic liquids containing mobile ionic or inorganic components and concluded that such membranes might respond to changes in external solution composition. Liquid membrane sensors for calcium ions were introduced in 1967 by Ross [38] and many others soon followed.

The idea of using a glass electrode for the sensing of gases originated in 1958 with Severinghaus [39] who coupled a CO_2_ diffusion membrane to the glass electrode. This has stimulated several other sensors for gases which affect the pH of water, as well as composite electrodes consisting of a primary detector electrode coupled with some sort of specific generating system. The nature of the generating system has been varied widely. The earliest seems to be the enzyme-substrate system urease-urea, sensed by Guilbault and Montalvo [40] in 1970 with a glass electrode to detect ammonium ions for measuring urea. Rechnitz [41] has been especially active in this field, devising not only enzyme-based electrodes, but electrodes based on antigen-antibody interactions, and sensors using plant or animal tissue membranes and even living organisms.

A different principle used for membrane sensors was introduced in 1956 when L. C. Clark [42] used a diffusion membrane to obtain stable diffusion-limited electrolysis currents at stationary electrodes. The first application, for monitoring dissolved oxygen, was soon commercialized but its obscure publication delayed the further exploitation of this principle.

b. Electrolytic techniques shifted away from classical polarography to other microelectrode techniques. Several reasons for this shift of emphasis can be traced in retrospect: (a) While diffusion theory to the dropping electrode was understood relatively early (Ilkovic, 1934) [43], many phenomena related to electrode kinetics remained ill-understood until Koutecky in 1953 [44] showed the complex relationship between diffusion and kinetics at the dropping electrode. (b) Instrumentation for current-time-potential measurements was primitive until the 1940s and later. Transient and pulse techniques lent themselves more simply to stationary electrodes than to the dropping electrode. (c) The development of the rotating disk and the ring disk electrodes by Levich in the 1940s provided an accurate means of defining mass transport and for studying transitory intermediates formed at electrodes.

Classical polarography played a key historic role in leading to the development of various microelectrode methods. A good example is chronopotentiometry, or measurement of transition times during electrolysis at constant current under diffusion control. The theory of transition times dates back to Sand (1901) [45] who verified the equation for long transition times with special precautions to avoid connective disturbances. Gierst and Juliard in 1953 [46] used a slowly dropping electrode to verify Sand’s equation for short transition times, thus illustrating the stimulating effect of polarography on other microelectrode techniques. Chronopotentiometry is also an example of a rapid rise and fall of a technique. After a flurry of papers in the 1950s and early 60s, it became recognized that the theoretical difficulties of eliminating charging currents limited the accuracy of the technique, which now is largely used for diagnostic purposes such as determining whether a soluble or insoluble product is formed. Pulse polarography, which grew out of classical polarography, is another example. In its first version by Barker [47] it was shown to be far more sensitive than the classical method, but it did not gain wide use because of its electronic complexity until greatly simplified versions were described by Parry and Osteryoung [48] and developed commercially by PAR. Ultramicroelectrodes have been found to have theoretical and practical advantages which require modern measurement techniques for their full realization. New electrode materials and modified electrode surfaces are enlarging the horizons of electroanalysis. Another capability of modern instrumentation is to use a variety of different applied signals and output measurements on a given cell setup to permit signal storage and retrieval.

c. Combinations of optical and electrochemical techniques such as electrochemiluminescence, and combinations of electrochemical sensors with separation techniques such as HPLC, are finding applications, especially in bioanalytical chemistry. The concept of fiber optic sensors goes back to 1976 [49], when they were suggested for monitoring a number of physical properties. The name “optrodes” and the idea of chemical sensing originated in 1983 with Thomas Hirschfeld et al. [50]. Although electroanalytical techniques are often fully competitive in sensitivity with spectrochemical methods and cheaper in instrumentation, they often fail to be considered because they may be more limited in scope and more demanding in knowledge of solution chemistry.

Curiously enough, the atomic absorption method did not emerge until 1955. The hollow cathode tube of Walsh was largely responsible for its spectacular rise thereafter. Another factor, however, was that the need for trace analytical data increased greatly during the 1950s and 1960s. For example, environmental chemistry stimulated interest in measuring pollutants, and increasing awareness of the effects of trace constituents in materials such as alloys and solid state electronics materials provided an enormous stimulus.

The technique of atomic fluorescence spectroscopy was suggested by Alkemade in 1962 and introduced analytically by Winefordner, who remarked in 1976 “The method has not become popular despite significant advantages over atomic absorption in some cases. The reasons are not very clear. Lack of commercial instrumentation may be part of the explanation, but more likely it is the overwhelming popularity of atomic absorption methods. Atomic fluorescence has not yet made it into the club” [7]. Ten years later, he said that his statement is still true despite the introduction of a commercial instrument. An added reason is the emergence of plasma emission sources, especially the inductively coupled plasma or ICP, which have become increasingly important in recent years. Introduced simultaneously by Fassel and by Greenfield in 1964, it has been intensively investigated by Fassel more recently. Being commercially available and applicable to multi-element analyses of great sensitivity, ICP spectroscopy has become the most important of present-day emission spectrochemical methods.

Chemiluminescence has been known since the 19th century but only in recent years has it seen extensive use in trace analysis because of a lack of selectivity. By controlling the reaction conditions and by improved instrumentation it is now possible to determine many substances including trace metals, oxidizing and reducing gases, and biochemicals by direct or indirect methods involving chemiluminescence. The measurements are usually transient in character so they require advanced instrumentation for their full exploitation. No doubt this is the reason for the slow development of this method. Another factor is the need for careful consideration of the chemical reactions involved.

The use of catalyzed reactions, and of kinetic methods in general was delayed until the development of instrumentation made possible the convenient measurement of reaction rates, even though the theoretical basis of such methods was well understood. For example, glucose oxidase has been used as a specific catalyst since 1957 [52] for the determination of glucose in blood serum via the production of hydrogen peroxide which reacts with a dye to form a colored reaction product. By designing an instrument for the automatic measurement of the initial reaction rate, Malmstadt and Hicks [53] in 1960 described a refined and specific method for glucose.

A different type of application of enzymes arose with the use of immobilized enzymes at an electrode surface. The first use of such an immobilized enzyme appears to be that of Clark and Lyons [54] in 1962, who immobilized glucose oxidase at a membrane-coated electrode and sensed the hydrogen peroxide amperometrically. The potentiometric enzyme electrodes have been mentioned above.

During the 1960s the development of the microcomputer made it possible to automate the measurement of the initial reaction rate by using the small change in voltage output of a transducer to register a small change in concentration. The transducer could be based on various principles, such as measurement of potential, current, or absorbance. Although some notable examples of trace analysis by non-catalytic methods have been described, the vast majority of kinetic trace methods are based on catalytic reactions.

Nuclear methods have existed in principle since the discovery of radioactivity at the turn of the century. Tracer techniques using naturally occurring isotopes date back at least to 1919, when Paneth used thorium B, a naturally occurring isotope of lead as a tracer to study the reactions of lead. With the discovery of artificial radioactivity in 1934, tracer techniques became more general. By the late 1930s Kolthoff was using radioactive bromine, prepared by using a radon-beryllium neutron source to study the aging of silver bromide [55]. Activation analysis dates back to 1938, when Seaborg and Livingood determined gallium in iron at the 6 ppm level using a cyclotron source. However, it was not until the nuclear reactor was available as a high flux neutron source in 1946 that neutron activation analysis became an important trace technique. Radioactive tracer isotopes as well as enriched stable isotopes soon became available for many applications of isotope dilution analysis.

The gas chromatographic method had been mentioned in a 1941 publication [56] but it lay dormant for 10 years before being revived by the same worker, A. J. P. Martin [57]. Liquid-liquid partition chromatography actually did see limited applications during the 1940s, and the Craig countercurrent extraction method was widely used in biochemical laboratories, but GC escaped attention. As it happened, Martin and Synge were engaged in unrelated wartime research soon after the 1941 publication, and it was not until 1948 when Martin and James resumed work on the idea. No doubt a contributing factor to this long neglect was the fact that the original publication in *Biochemical Journal* was not available during the war in many countries, and in any case, this journal was not one that most analytical chemists would consult. Soon after the publication of several papers in the early 1950s, commercial development followed. In the U.S., apparently the first GC was built at Monsanto by Ralph Munch in 1953 and described at a Gordon Conference in 1954. It used a thermal conductivity detector. By 1955 commercial equipment was available. Many of the early applications were for major and minor constituents in mixtures and for analysis of small samples, but later, trace methods became important, especially after the introduction of more sensitive detectors such as the flame ionization detector in 1958. About the same time, the open tubular column or capillary column GC, and GC-MS were developed, both of which greatly expanded trace applications. Commercial GC-MS did not become available until around 1970, because of the need for small dedicated computers to handle the voluminous amount of data.

The introduction of ion chromatography by Small et al. [58] in 1975 as a special form of ion exchange represented an important advance in trace analysis especially for anions but also for mixtures of cations. For extremely dilute samples, a preconcentration step can easily be added to collect ions from a large sample onto a precolumn, from which they are eluted into a separation column.

Liquid chromatography underwent a revolution starting in 1967 with the introduction of the Waters ALC 100 high pressure liquid chromatography system. The letters HPLC were first used to designate high pressure liquid chromatography and later high performance liquid chromatography. Coupling of LC with MS began in 1973–74 with the publication of results from the laboratories of E. C. Horning, F. W. McLafferty, and R. P. W. Scott. However, early efforts were fraught with difficulties due to the need for removing the solvent, and even today improvements are being actively pursued.

Mass spectrometry has continued to develop in several forms as an important trace analytical method. Spark source MS dates back in principle to Dempster in 1934 but modern instrumentation and quantitation did not come about until the 1950s. Time of flight MS originated with A. E. Cameron in 1948 and was commercialized by Bendix in 1955. Secondary ion MS, using an ion beam to sputter material from a solid surface, emerged in the 1960s and is important both in the imaging and ion probe configurations. Tandem mass spectrometry (MS/MS) was first introduced as a structural tool in the mid-1960s but it did not become important as a trace analytical method until the triple quadrupole system of the late 1970s. The quadrupole MS dates back to Paul and Rather in 1955, and became commercially available during the 1960s. The triple QMS system, developed by Yost and Enke in 1978 and commercially available since 1981, is becoming increasingly important in trace analysis, especially for complex organic mixtures. Still another variant is ICR MS, or ion cyclotron resonance mass spectrometry, which originated in principle as the Omegatron at NBS in 1950, and which became commercially available from Varian in 1967. The Fourier transform version, introduced in 1974, has improved its applicability to analytical problems, but it is still not primarily a trace analytical instrument.

X-ray emission is another trace analytical method that was slow to develop. The principles were known at the time of Moseley (1913), who discovered the concept of atomic numbers, but analytical applications were slow to emerge. Birks, who with Friedman introduced the modern version of the x-ray fluorescence in 1948, has traced the slow development of the method over the intervening 35-year period [7]. In 1914, de Broglie had demonstrated the excitation of fluorescence x-rays outside the x-ray tube; Jonsson in 1927 had made accurate intensity measurements by means of a Geiger counter; von Hevesy published a book in 1932 laying out the principles of x-ray emission analysis; and a Russian book by Borovskii and Blokhin in 1939 formed the basis of a course on the subject at Moscow University. However, in the period between 1932 and 1948, hardly any publications appeared on the subject. Birks remarks that the modern development “was not deliberate but rather the result of a chance observation of strong background interference in x-ray powder diffraction patterns of Fe compounds when using a Cu target x-ray tube.” “Changing to an Fe-target tube eliminated the background difficulties, but Friedman recognized the potential of using the fluorescent excitation as a means of elemental analysis.” Birks, in following the later development of x-ray emission through the 1950s to the 70s, states that we “observe that its success depended not on new x-ray principles but almost entirely on various kinds of improvements in electronics.”

The electron microprobe, also based on x-ray emission, was patented in 1947 by Hillier of RCA in 1947 but he did not pursue the method and Guinier and Castaing reported on their conversion of the electron microscope to a microprobe in 1949. An independent development by Borovskii in the U.S.S.R. occurred about the same time. The microprobe was of special significance because it permitted the direct observation of spatial distribution of constituents that on the basis of average composition would be trace constituents but would sometimes have pronounced effects because of segregation in regions of higher concentration such as grain boundaries. A closely related technique allowing for finer spatial resolution at the expense of selectivity and sensitivity is the use of energy-dispersive x-ray analysis of surfaces with the scanning electron microscope, developed during the 1960s.

Another important x-ray technique is x-ray photoelectron spectroscopy (XPS or ESCA), developed in the late 1960s by Siegbahn. Analytical applications were greatly stimulated by the publication of a monograph in 1967 and by the introduction of commercial instrumentation in 1970. More recently, the trend has been to incorporate ESCA measurements with other ultrahigh vacuum spectroscopy techniques involving a number of different excitations (photon, ion, or electron) and various types of signal (photon, ion, or electron). Although these are primarily surface techniques where the local concentration is not necessarily at the trace level, the extreme sensitivity of the techniques suggests their inclusion in any discussion of trace analysis. Ion etching of the surface permits probing of composition into the third dimension.

From these historical examples it is clear that long delays often occurred between the discovery of a trace method and its practical application. Several reasons for these delays can be found in retrospect. Sometimes, as in the case of amperometric titrations and voltammetry, the theory was ill-understood at the time of the original discovery. In other cases, e.g., gas chromatography, the seminal publication did not reach the proper readership for a long time. In still other cases, as in chronopotentiometry, the basic theory was well known but the measurements could not be put to practice because of the lack of instrumentation. Another factor was that certain types of measurements required special materials for their exploitation, as in the case of thermometric measurements which were facilitated by the development of the thermistor. Finally a need must exist before widespread use of a method will occur. Oftentimes more than one of these factors came into play, as in the case of atomic emission spectroscopy.

If we look at trace analytical methods as a group, we find a parallelism during the past 50 years regardless of the early history. In the first place there was not a great emphasis on quantitative applications of trace methods until the need arose, so many fundamental findings tended to lie stagnant until about 1940. Secondly, instrumentation was relatively primitive until the 1930s when electronics began to play a significant role. Electronic instrumentation was to undergo two major leaps ahead, first the change from vacuum tube to solid state electronics, and second with the emergence of the microcomputer, which became small enough and inexpensive enough to become an integral part of the measurement system. The availability of commercial instrumentation has also been an important factor in the use of a particular approach.

It may also be relevant to consider the reasons for the popularity of certain trace methods in relation to others. Among the factors that determine which method is to be used for a given problem (given that the sensitivity is adequate) are the following:

- Type of sample. Solid samples may be analyzed directly by some methods but require dissolution for others. The dissolution process may be so time consuming as to rule against the use of some methods. Similarly, gaseous samples and liquid samples may be directly analyzed by some methods but require conversion in others.
- Homogeneity of samples. Some methods inherently examine extremely small samples, and therefore are more susceptible to sampling errors if the average composition is sought. Conversely, if distribution is to be studied, a method of sufficient spatial resolution is required.
- Cost of equipment. Some methods require enormous capital investments, which may or may not be justified by the nature of the problem.
- Cost of personnel. Some methods are easily converted to a routine procedure that gives reliable results in the hands of a technician trained specifically for that procedure, while other methods inherently involve adjustments depending on the sample and therefore need personnel of greater training. This factor is important, for example in the relatively great usage of spectroscopic methods for trace metal determination as compared with electrochemical methods, which involve much lower investments in capital equipment.
- Type of information needed. If just elemental composition is required, it is better to use a method insensitive to the chemical state of the elements. By the same token, such a method will not give information as to the chemical state, if that information is required. Neutron activation analysis is a good example of a method insensitive to chemical state, while electrochemical methods do respond to chemical state, and therefore provide information of this type.
- Speed. Some problems require rapid response, and therefore require a method of appropriate speed. Continuous analysis of a flowing stream represents a problem requiring a rapid response time.
- Dynamic range. Some methods will respond to samples ranging widely in composition, while others need a prior adjustment by dilution or concentration to bring samples into their dynamic range.
- Selectivity. Some methods are subject to numerous interferences unless provision is made for prior separations. Nevertheless, if the interferences are known to be absent, such a method may find uses in special situations.
- Number of samples to be analyzed. The analysis of a single sample may lead to a different choice of method than the analysis of a series of samples. Furthermore, the chosen method may be different in different laboratories depending on the available equipment and personnel.

It has been my purpose to discuss the origins of a variety of trace analytical methods and to examine the reasons for the sometimes halting nature of their development into modern form. It would also be proper to consider recent trends, which are a useful guide to future expectations, and to make a critical comparison of the various trace analytical methods. However, there are to be two talks on the present day status of trace analysis, so these topics are better left for my colleagues.
